# Molecular Characterization of *Lipoptena* *fortisetosa* from Environmental Samples Collected in North-Eastern Poland

**DOI:** 10.3390/ani11041093

**Published:** 2021-04-12

**Authors:** Remigiusz Gałęcki, Xuenan Xuan, Tadeusz Bakuła, Jerzy Jaroszewski

**Affiliations:** 1 Department of Veterinary Prevention and Feed Hygiene, Faculty of Veterinary Medicine, University of Warmia and Mazury in Olsztyn, 10-719 Olsztyn, Poland; bakta@uwm.edu.pl; 2 National Research Center for Protozoan Diseases, Obihiro University of Agriculture and Veterinary Medicine, Obihiro 080-8555, Japan; gen@obihiro.ac.jp; 3 Department of Pharmacology and Toxicology, Faculty of Veterinary Medicine, University of Warmia and Mazury in Olsztyn, 10-719 Olsztyn, Poland; jerzyj@uwm.edu.pl

**Keywords:** deer keds, ectoparasite, Hippoboscidae, PCR, phylogenetics, vector

## Abstract

**Simple Summary:**

*Lipoptenafortisetosa* is an invasive, hematophagous insect, which lives on cervids and continues to spread across Europe. The species originated from the Far East and eastern Siberia. Besides wild animals, these ectoparasites can attack humans, companion animals, and livestock. These insects may also play a role in transmitting infectious diseases. The objective of this study was to confirm the presence of *L.* *fortisetosa* in north-eastern Poland and to characterize the examined population with the use of molecular methods. Deer keds were collected from six natural forests in the region of Warmia and Mazury. DNA of *L.* *fortisetosa* was extracted and subjected to molecular studies. Two species of deer keds (*Lipoptena* *cervi* and *L.* *fortisetosa*) were obtained in each location during field research. There were no differences in the sex distribution of these two ectoparasite species. During the research, more *L.* *cervi* than *L.* *fortisetosa* specimens were obtained. The studied insects were very closely related to specimens from Lithuania, the Czech Republic, and Japan. Our study indicates various ectoparasite lineages, and such research contributes to the improvement of basic knowledge on the distribution, genetic structure, and variability of the invasive ked fly *L. fortisetosa*.

**Abstract:**

Recent years have witnessed an increase in the population of *Lipoptena* *fortisetosa* in Central Europe. The genetic profile of this ectoparasite has not been studied in Poland to date. The aim of the present study was to confirm the presence of *L.* *fortisetosa* in north-eastern Poland and to characterize the examined population with the use of molecular methods. Deer keds were collected between June and July 2019 in six natural, mixed forests. A fragment of the rRNA 16S gene was used as a marker to identify *L.* *fortisetosa* by polymerase chain reaction (PCR). DNA samples were sequenced in the last step. Six new locations of *L. fortisetosa* were confirmed. No significant differences were observed in the sex ratios of *L. cervi* and *L. fortisetosa* (*L. cervi p*-value = 0.74; *L. fortisetosa p*-value = 0.65). Significant differences were noted between the total size of *L. cervi* and *L. fortisetosa* populations (*p*-value < 0.001). The similarity to GenBank sequences ranged from 95.56% to 100%. The obtained nucleotide sequences were very closely related to *L. fortisetosa* sequences from Lithuania, the Czech Republic, and Japan. Molecular analyses revealed considerable genetic diversity, which could indicate that various ectoparasite lineages have spread throughout Europe.

## 1. Introduction

Deer keds are hematophagous ectoparasites of birds and mammals. The genus *Lipoptena* consists of 32 species, including *L. capreoli*, *L. cervi*, *L. depressa*, *L. mazamae*, and *L. fortisetosa*, which are of great importance in veterinary medicine. *Lipoptena fortisetosa* has been insufficiently investigated in cervids, despite the Japanese sika deer (*Cervus nippon*) that is its original host. The parasite also colonizes non-specific hosts, including cattle and humans [1,2]. Deer keds shed wings when they find a definitive host. Hippoboscidae reproduce by adenotrophic viviparity, and their life cycle is influenced mainly by climatic conditions [3,4]. *Lipoptena* spp. bites are painful and may lead to skin inflammation and alopecia [5,6].

There is evidence to suggest that *L. fortisetosa* continues to spread across Europe. The species originated in the Eastern Palearctic, in particular, the Far East and eastern Siberia. *Lipoptena fortisetosa* was first identified in 1965 in Japan by Maa [7]. In Central Europe, this deer ked is considered a cryptogenic species, but it is increasingly classified as an invasive species [8]. *Lipoptena fortisetosa* was first recorded in Europe in the 1960s [9]. Currently, this insect was spotted in several European countries including the Czech Republic [10], Estonia [11], Italy [12,13], Moldova [2], and Slovakia [14]. This ectoparasite was not detected in Poland during a program monitoring the ectoparasite burden of wild animals in 1973–1980 [15]. In Poland, *L. fortisetosa* was first observed in the region of Lower Silesia in the late 1980s [16]. Later, in 2007–2012, *L. fortisetosa* was identified in cervids in northern Poland and in the Tatra Mountains [8,17,18,19,20]. In 2017, this ectoparasite was found even in companion animals in central Poland [21].

*Lipoptena fortisetosa* is a potential vector of infectious diseases. Deer keds can act as carriers of *Bartonella* spp., and the vertical transmission of this pathogen poses a particularly serious problem [22,23,24]. In recent research, *Bartonella* spp. of Japanese origin were isolated from *L. cervi* in the region of Warmia and Mazury in Poland [22]. According to Szewczyk et al. [22], the isolated *Bartonella* spp. strain had originated from Japanese sika deer that were introduced to Europe, and it was transmitted to the local environment by various vectors. Molecular studies confirmed that the insects harbored genetic material of *Anaplasma phagocytophilum*, *A. ovis*, *Borrelia* spp., *Coxiella*-like bacteria, *Theileria luwenshuni*, *T. ovis*, and *Trypanosoma* spp. [25,26,27,28,29,30,31]. Despite the above, little is known about the ectoparasite’s ability to transmit pathogens other than *Bartonella* spp. to new hosts.

The observed migration patterns of the analyzed ectoparasites suggest that their geographic range should be monitored because of the potential vectorial capacity of this ectoparasite [32]. Salvetti et al. [12] noted that the geographic distribution of deer keds in Europe should be analyzed for social and environmental implications because *Lipoptena* spp. may cause problems for wildlife management. However, a detailed evaluation of ancestral lineages and migration routes requires larger and more frequent sampling. There is a considerable gap in the genetic distribution of *L. fortisetosa* between Western and Eastern Europe. Andreani et al. [33] did not detect any obvious and stable morphological and molecular differences in specimens from the geographical areas of Asia and Europe. In Italy, they identified the monophyletic clade of *L. fortisetosa*, along with two Central European and two Korean haplotypes [33]. These ectoparasites have been largely neglected in Polish research, and potentially important genetic data, including genetic diversity, are scarce. Most Polish studies of *L. fortisetosa* were based on macroscopic analyses, which is why the genetic and phylogenetic characteristics of the species, including DNA rRNA 16S gene remain unknown. The locations colonized by *L. fortisetosa* in north-eastern Poland have not been evaluated to date [4]. This Polish region abounds in forests, and it is characterized by increasingly frequent deer ked flights and mass attacks on animals and humans. Differences in sex ratios of host-seeking insects were not investigated in this location. This may be important because of the fact that females are more likely to harbor the genetic material of pathogens [32]. The spread of *L. fortisetosa* outside of Asia could have been accelerated by the introduction of sika deer to the European continent, whose small but stable populations can be found in the central part of the region of Silesia and in the eastern part of the region of Warmia and Mazury. The aim of this study was to confirm the occurrence of *L. fortisetosa* in north-eastern Poland and to characterize the specimens collected during host-seeking flights based on the results of molecular analyses.

## 2. Materials and Methods

### 2.1. Sample Collection

**Figure 1:**
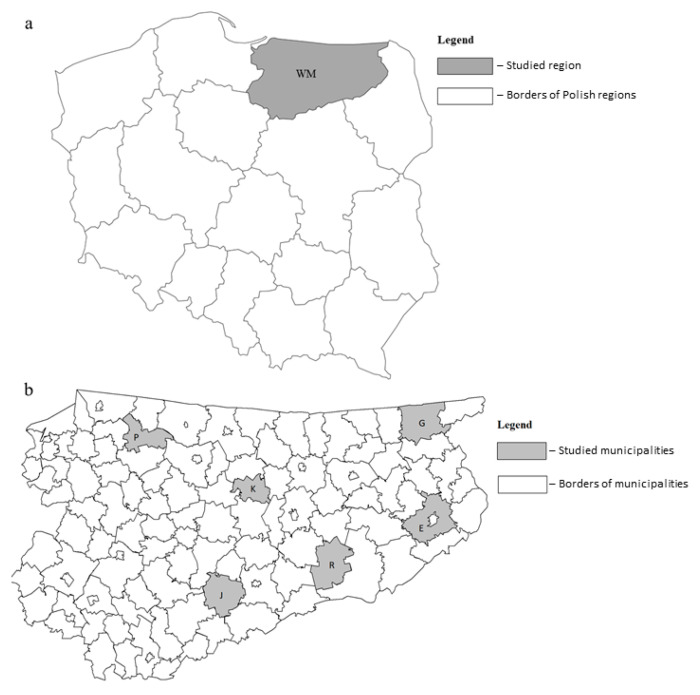
(**a**) Map of Poland and the region of Warmia and Mazury (**b**) with an indication of the municipalities were field studies were performed. Legend: WM = the region of Warmia and Mazury; E = Ełk; G = Gołdap; J = Jedwabno; K = Kolno; P = Pieniężno; R = Ruciane-Nida.

The occurrence of *Lipoptena* spp. was monitored in natural mixed forests in the region of Warmia and Mazury in north-eastern Poland. Ectoparasites were monitored in the following municipalities: Ełk (54°19′10.1″ N 22°22′38.7″ E), Gołdap (54°19′10.1″ N 22°22′38.7″ E), Kolno (53°57′34.3″ N 21°04′46.0″ E), Jedwabno (53°28′24.8″ N 20°34′04.9″ E), Pieniężno (54°15′52.1″ N 20°11′14.2″ E), and Ruciane-Nida (53°35′47.4″ N 21°33′13.3″ E). A detailed map of the surveyed region is presented in Figure 1.

The selected sampling sites are characterized by extensive forests and large cervid populations, including red deer (*Cervus elaphus*), European roe deer (*Capreolus capreolus*), and moose (*Alces alces*). The study was conducted between June and July 2019 between 7 and 10 a.m. This study period was chosen because of a noticeable increase in hippoboscidae activity. The number of *Lipoptena* spp. individuals was expressed by the sum number of winged deer keds that were captured on the investigator’s clothing per day. Five daily measurements were conducted in each sampling site. A total of 30 measurements were conducted. The investigator walked through the forest wearing brown cotton clothing covering the entire body. *Lipoptena* spp. were captured immediately after landing on the clothing. During the walk, the investigator checked protective clothing every 20 m and collected insects from creases and folds on the fabric. Keds that escaped were not counted. Similar methods have been used by other researchers to acquire environmental samples of ticks and *Lipoptena* spp. [34,35]. The collected ectoparasites were placed in individual test tubes containing 70% ethanol.

### 2.2. Species Identification

The collected samples were transported to the Biological Hazard Laboratory at the Faculty of Veterinary Medicine of the University of Warmia and Mazury in Olsztyn. Species and sex were identified based on morphological features, including body dimensions, wing venation, the length and structure of the palpi, and the number of erect hairs on the mesonotum, under the Leica M165C stereoscopic microscope (Leica, Wetzlar, Germany). Measurements were performed in Leica Application Suite 4.4 (Leica, Wetzlar, Germany) based on the taxonomic keys for *Lipoptena* spp. developed by Borowiec and Maa, and the morphological descriptions provided by Andreani et al. [13,36,37].

### 2.3. Statistical Analysis

The sex ratio of *Lipoptena* spp. and the number of *L. cervi* and *L. fortisetosa* specimens from each measurement were analyzed with Student’s t-test for independent samples. The following statistical parameters were calculated for the analyzed populations of *L. cervi* and *L. fortisetosa* using overall data from daily observations (n = 30): mean (*M*), median (*ME*), standard deviation (*SD*), standard error (*SE*), variance (*V*), minimum (*Min*), and maximum (*Max*). Differences were regarded as statistically significant at *p*-value < 0.05. Data were processed statistically in Statistica 13.3 (TIBCO Software Inc., Palo Alto, CA, USA).

### 2.4. DNA Extraction

Five samples of *L. fortisetosa* (n = 30) from each sampling site were randomly selected for molecular analyses. The representative number of samples was estimated with the use of formulas for computing the minimum sample size to guarantee the reliability of the results (sample size: *N_P_* = number of collected specimens, *α* = 95%, *f* = 0.5, *e* = 15%). Keds were air-dried at room temperature for 15 min, and they were crushed with a sterile rod in sterile Eppendorf tubes. Genomic DNA was extracted from each sample with the Sherlock AX kit (A&A Biotechnology, Poland) according to the manufacturer’s instructions. DNA was eluted in 40 μL of Tris-EDTA (TE) buffer, and the concentration of the extract was checked in the Nano Drop 2000 spectrophotometer (Thermo Fisher Scientific, Waltham, MA, USA). The extracted DNA was stored at −20 °C until analysis.

### 2.5. Polymerase Chain Reaction

The DNA sequence of the mitochondrial DNA rRNA 16S gene of *L. fortisetosa* with an estimated length of 412-bp was amplified by PCR. The amplification primers were L700F (5′-AAAGTTTAACCTGCCCACTGAT-3′) and L1213R (5′-CTGAACTCAGATCACGTAAGAAT-3′) [22]. The following cycling conditions were applied: initial denaturation at 92 °C for 3 min; 35 cycles of denaturation at 95 °C for 10 s; annealing at 60 °C for 10 s; extension at 68 °C for 30 s; final extension at 68 °C for 5 min [22]. Each reaction was performed in a final volume of 25 μL containing 2.5 μL of 10× Standard Taq Reaction Buffer (Biolabs, NC, USA), 0.5 μL of 10 mM deoxyribonucleotide triphosphate (dNTPs) (Biolabs, USA), 0.5 μL of 10 μM of each primer, 1 μL of *Lipoptena* spp. DNA template, 0.125 μL of Taq DNA Polymerase (Biolabs, NC, USA), and 19.875 μL of double-distilled water. Double-distilled water was the negative control. *Lipoptena cervi* DNA extracted during a previous study was the positive control. PCR products were subjected to electrophoresis on 2% agarose gel. They were stained with ethidium bromide and visualized in a UV transilluminator.

### 2.6. Sequencing

After PCR, DNA samples were purified by the ethanol precipitation method described by Weinberger [38]. DNA was sequenced with the use of the described primers (L700F/L1213R) and the BigDye Terminator Cycle Sequencing Kit (Applied Biosystems, Foster City, CA, USA), and the results were analyzed in the ABI PRISM 3100 Genetic Analyzer (Applied Biosystems, Foster City, CA, USA). The acquired nucleotide sequences were edited in the BioEdit program [39] and compared with GenBank sequences in the BLAST-NCBI program (attached in result section). Sequences were aligned with the ClustalW algorithm. A phylogenetic analysis of rRNA 16S sequences and the corresponding GenBank sequences was performed by the maximum likelihood method and the Tamura–Nei model in MEGA 10.1.17 [40]. Sequences of *L. cervi* served as the outgroup. Model selection was based on Akaike’s information criterion (AIC). Bootstrap confidence values for assessing the reliability of branches were calculated in 10,000 replicates.

## 3. Results

The representative *L. fortisetosa* sequences were deposited in the GenBank database of the National Center for Biotechnology with the BankIt tool under accession numbers MT182645-MT182661, MT182663.

A total of 482 *Lipoptena* spp. specimens (*M* = 80.33; *ME* = 79.5; *SD* = 12.39; *SE* = 5.06; *V* = 15.43; *Min* = 61; *Max* = 96) were collected in sampling sites. Of those, 385 were identified as *L. cervi* (*M* = 64.17; *ME* = 67; *SD* = 9.26; *SE* = 3.78; *V* = 14.43; *Min* = 47; *Max* = 72), including 196 females (50.9%) and 189 males (49.1%). No significant differences in the sex ratio were noted in *L. cervi* (*p*-value = 0.74). *Lipoptena fortisetosa* was recorded in all six locations. Ninety-seven individuals were identified as *L. fortisetosa* (*M* = 16.17; *ME* = 15.5; *SD* = 5.53; *SE* = 2.56; *V* = 34.20; *Min* = 8; *Max* = 24), including 51 females (52.58%) and 46 males (47.42%). No significant differences in the sex ratio were found (*p*-value = 0.65). The overall populations of *L. cervi* and *L. fortisetosa* from studied locations differed significantly in size (*p*-value < 0.001). The results are presented in detail in Table 1.

**Figure 2:**
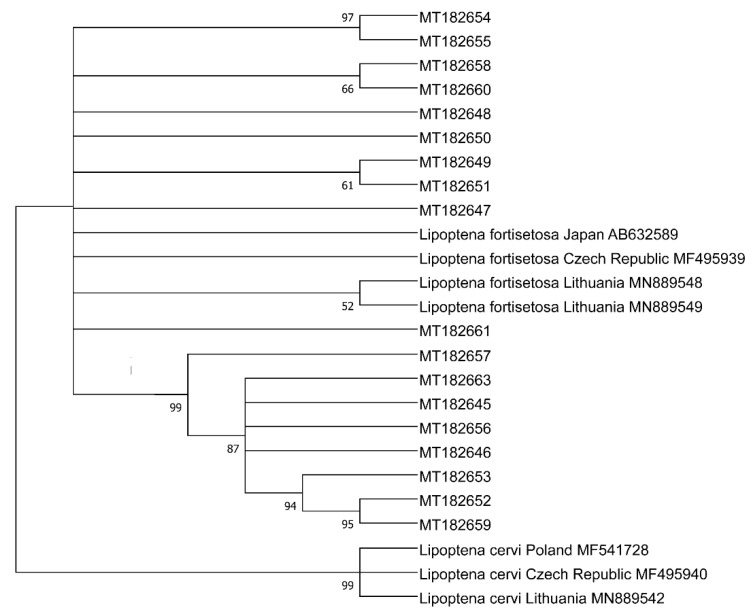
Phylogenetic analysis of the rRNA 16S gene in *Lipoptena fortisetosa*. Legend: phylogenetic tree based on the results of the maximum likelihood analysis of a fragment of the rRNA 16S gene in *Lipoptena fortisetosa*. The identified unique haplotypes are labeled with sequence identification numbers. The reference sequences from GenBank are indicated in the tree. Bootstrap confidence values for assessing the reliability of branches were calculated in 10,000 replicates.

The phylogenetic tree is presented in Figure 2. The length of the alignment used to build the phylogeny was 445-bp. Twenty-eight sequences (MT182645-MT182658, MT182660-MT182661, MT182663) were characterized by 98.32% to 100% similarity with *L. fortisetosa* sequences from the Czech Republic and Lithuania (MF495939, MN889549). The similarity between sequences MT182659 and the *L. fortisetosa* sequence from Japan (AB632589) reached 95.56%. Detailed data, including the ID of the closest match, country of origin, and percent sequence identity, are presented in Table 2. The distribution of sequences in each sampling site is presented in Table 3.

## 4. Discussion

Autochthonous *L. cervi* accounted for 79.88% of the collected ked samples, which suggests that it is the dominant species in the region of Warmia and Mazury. However, the proportions of *Lipoptena* species should be regularly monitored in the studied region because of the possible increase in the population of *L. fortisetosa*. In Europe, *L. fortisetosa* is considered a multivoltine species, whereas *L. cervi* is regarded as a univoltine species [8]. The phenomena described above could influence the rate at which these ectoparasite populations develop in the future, and it could increase the number of host-seeking *L. fortisetosa*. Because of phenological differences between the species, the number of collected individuals can also be influenced by the month of sampling. Both ectoparasites species should be sampled in a period when they are most active. This study demonstrated that adult *L. fortisetosa* and *L. cervi* are active in the same period of time, and similar observations were made by Oboňa et al. [14]. Neither species was characterized by significant differences in the sex ratio. In around 30% of ectoparasitic species, the sex ratio does not significantly depart from unity [44]. Adult keds find hosts immediately after emergence. However, the sex ratio of *Lipoptena*-spp.-inhabiting hosts could differ from that noted in this study because of a potentially shorter life span of adult males [44].

The migration patterns of *Lipoptena* spp. in Europe have not been fully elucidated, but the geographic range of these ectoparasites was probably expanded by natural migration from the east. It cannot be ruled out that this insect may have been brought to Europe along with imported animals [33]. *Lipoptena fortisetosa* had been previously identified in only several European countries, including the Czech Republic [10], Moldova [2], and Poland [4,8]. Recent reports from Estonia [11] and Italy [12,13,33] indicate that the geographic range of *Lipoptena* spp. has expanded both northward and southward since 2017. Andreani et al. [33] suggested that the relatively recent migration of sika or hybrid individuals to Italy from neighboring countries may have contributed to the ectoparasite transmission. There is no conclusive evidence to indicate whether this geographic shift has resulted from an increase in the cervid population or climate change. The impact of global climate change on the geographic expansion of *Lipoptena* spp. was postulated by Kurina et al. [11]. Keds are poor fliers that cover a distance of only up to 50 m when searching for hosts [45], which is why hosts play an important role in the transmission of these ectoparasites. The presence of *L. fortisetosa* in highly diverse regions also suggests that these insects are eurytopic species that tolerate a wide range of environmental conditions [4,46].

The importance of *Lipoptena* spp. in veterinary medicine has not been fully established, which is why these ectoparasites should be studied in greater detail. The existing knowledge about the impact of *L. fortisetosa* on wild animals is superficial, and these ectoparasites could affect game management in the future. Polish cervids and other wild animals were found to be heavily infested with hippoboscids. Deer keds were detected in 64% of European roe deer [47], 76% of fallow deer, and 78% of European red deer [48]. One animal was infested with 9.9 insects on average [48]. Vikøren et al. [49] found that one moose can carry more than 16,000 keds. Andreani et al. [33] detected infestation of European deer species by *L. fortisetosa*. The stability of the *L. fortisetosa* population can be attributed to adaptive genetic variation and the benefits associated with colonizing different cervid species as hosts. These observations point to high plasticity in host selection, which is influenced by the availability and population density of a given host species. For this reason, there are no obvious barriers to the future expansion of the discussed ectoparasites [11,33].

The available GenBank data show that the sequences originating from the Czech Republic or Lithuania (cited in the manuscript) were closely similar to ours. However, the GenBank database contains only several 16S rRNA sequences of European *L. fortisetosa*. Sequence MT182659, obtained in the present study, was most similar to the sequence originating from Japan (AB632589) [43]. The insect harboring the above sequence was captured in the municipality of Pieniężno where a stable population of sika deer has existed since the beginning of the 20th century. *Lipoptena fortisetosa* could have been accidentally introduced to that region by sika deer, and a distinct population of these ectoparasites could have evolved over time. Similar conclusions were formulated by Andreani et al. [33] and Mihalca et al. [50]. Interestingly, two Korean haplotypes of this insect were detected in Italy [33]. This confirmed our assumptions. Polish deer keds could belong to an isolated and genetically diverse population. The genetic polymorphism of *L. fortisetosa* is probably associated with the segregation of haplotypes that are relatively popular in this species, and the stability of the *L. fortisetosa* population points to the presence of dynamic evolutionary mechanisms.

Similar levels of genetic diversity were reported in a study carried out on *L. mazamae* [51]. Trout et al. [51] identified six unique haplotypes using 30 DNA sequences of a 259-bp region of the mitochondrial DNA rRNA 16S gene amplified by PCR. Such molecular differences were also reported in other ectoparasites, including *Cimex lectularius*, tsetse flies (*Glossina* spp.), and ticks [52,53,54,55]. Szalanski et al. [52] observed high mitochondrial DNA diversity with moderate to high levels of gene flow in *C. lectularius*. Krafsur et al. [53] identified 26 haplotypes in tsetse flies with the use of 12S rRNA and 16S rRNA markers, where most haplotypes occurred only once or twice. Only six haplotypes were identified in the same locations [53]. 16S rRNA and Cytochrome c oxidase I (COI) gene sequences are also useful markers for analyzing genetic polymorphism in ticks and for identifying and differentiating the populations of *Dermacentor nuttalli*, *Ixodes persulcatus*, and *I. pavlovskyi* [54,55]. These findings indicate that the mitochondrial DNA rRNA 16S gene can be reliably used to study the population genetics of ectoparasites. Previous research has demonstrated that *Lipoptena* spp. could be paraphyletic; therefore, further research is needed to explore the genetic variation and taxonomy among this genus [56]. Kurina et al. [11] and Andreani [33] found that the COI sequences of *L. fortisetosa* deposited in GenBank are genetically diverse and have different geographic lineages in Europe. Similar conclusions can be formulated based on the results of this study.

The current study revealed considerable genetic diversity of *L. fortisetosa* in six sampling sites in the region of Warmia and Mazury, which indicates that the genetic structure of its population is far more complex than previously thought. Several genetically distinct original populations probably migrated or were introduced to Europe. The present findings suggest that a decrease in deer ked populations in forests could contribute to their diversity. The presence of progressive evolutionary mechanisms indicates that *L. fortisetosa* could further adapt to the environmental conditions of Central Europe [4]. *Lipoptena fortisetosa* is an invasive species that strongly competes for hosts with the native population of *L. cervi,* which could influence the size of both insect populations in the future. Further genetic analyses of louse flies, combined with pathogen detection, are needed to identify areas with a potentially high risk of infectious disease transmission by *L. fortisetosa* [51]. The structure of deer ked populations should be analyzed in greater detail to adequately manage and protect wild animals and to monitor the transmission of pathogens between distant regions. The identification of *Bartonella* spp. of Japanese origin by Szewczyk et al. [22] and *L. fortisetosa* of Japanese origin in this study demonstrates how the dispersion of deer keds in the environment can contribute to the spread of infectious diseases transmitted by these vectors. The results of this study provide additional evidence that *L. fortisetosa* populations in Central Europe have different genetic lineages. Detailed genetic characterization of deer keds in Central Europe will enable researchers to track and predict the spread of *L. fortisetosa*. Migrations to new areas should encourage new research into the dynamics and genetics of *L. fortisetosa* populations because, according to Kurina et al. [11], a thorough understanding of the distribution and bionomics of this invasive migratory species is crucial for its effective control in Europe.

## 5. Conclusions

The results of the present study and previous research suggest that *L. fortisetosa* has formed stable and genetically diverse populations throughout Poland. The population genetics of *L. fortisetosa* should be further explored to track the migration of these ectoparasites to Northern and Western Europe. The occurrence of *L. fortisetosa* should be regularly monitored to detect an increase in the number of individuals in the total deer ked population. Further research is needed to detect new locations and analyze the genetic lineages of *L. fortisetosa*. The sequences identified in this study indicate that the population of *L. fortisetosa* continues to spread despite geographic limitations.

## Figures and Tables

**Table 1:** Number of deer keds collected in sampling sites, with a division into species and sexes.

Sampling Site	*Lipoptena cervi*		*Lipoptena fortisetosa*		Total
	♂	♀	♂	♀	
Ełk	28	39	3	5	75
Gołdap	29	32	7	10	78
Jedwabno	34	33	6	8	81
Kolno	40	32	14	10	96
Pieniężno	20	27	7	7	61
Ruciane-Nida	38	33	9	11	91

Legend: ♂ = male; ♀ = female.

**Table 2:** Comparison of the acquired sequences with GenBank sequences.

Sequence ID	Closest Match ID	Country of Origin	Percentage Match	Reference
MT182645	MF495939 MN889549	Czech Republic Lithuania	99.56%	Radzijevskaja et al. [41] Sochova et al. [42]
MT182646			99.78%	
MT182647			98.68%	
MT182648			99.58%	
MT182649			99.36%	
MT182650			99.58%	
MT182651			100%	
MT182652			98.32%	
MT182653			99.04%	
MT182654			98.93%	
MT182655			98.93%	
MT182656			98.91%	
MT182657			98.43%	
MT182658			99.36%	
MT182659	AB632589	Japan	95.56 %	Hosokawa et al. [43]
MT182660	MF495939 MN889549	Czech Republic Lithuania	99.36%	Radzijevskaja et al. [41] Sochova et al. [42]
MT182661			99.15%	
MT182663			98.91%	

**Table 3:** Molecular characteristics of *Lipoptena fortisetosa* in sampling sites.

Number of *Lipoptena fortisetosa* Specimens						
Sampling Site	Ełk (n = 5)	Gołdap (n = 5)	Jedwabno (n = 5)	Kolno (n = 5)	Pieniężno (n = 5)	Ruciane-Nida (n = 5)
MT182645	2	-	-	-	-	-
MT182646	1	-	-	-	-	-
MT182647	1	1	-	1	-	-
MT182648	1	-	-	-	-	-
MT182649	-	1	-	-	-	-
MT182650	-	1	-	-	-	-
MT182651	-	1	-	1	-	-
MT182652	-	-	1	-	-	-
MT182653	-	-	1	-	-	2
MT182654	-	-	1	-	-	1
MT182655	-	-	1	-	-	1
MT182656	-	-	-	1	1	-
MT182657	-	1	-	1	-	-
MT182658	-	-	1	1	1	-
MT182659	-	-	-	-	1	-
MT182660	-	-	-	-	1	-
MT182661	-	-	-	-	1	-
MT182663	-	-	-	-	-	1

## Data Availability

All data generated or analyzed during this study are presented in this paper.
